# Vaccination in the Light of the Royal British Commission

**Published:** 1897-09

**Authors:** Alex. Wheeler

**Affiliations:** 37 Victoria Road, Darlington, England


### VACCINATION IN THE LIGHT OF THE ROYAL BRITISH COMMISSION.

37 Victoria Road,

DARLINGTON, ENGLAND, September 12th, 1897.

EDITOR OF THE HOMŒOPATHIC PHYSICIAN:

For many years I have worked, and suffered, in opposing vaccination. It has therefore been to me a very great delight to notice the enormous increase in the opposition to this rite in this country. But up to a recent time there has been but little to observe of opposition in the United States. Recently the articles in THE HOMŒOPATHIC PHYSICIAN have filled me with hope, and I now write to give you my most cordial thanks for thus opening a powerful medical paper to the discussion of the subject, and for permitting Dr. Leverson to inform your readers as to the doings of our Royal Commission. I hope that there will be a present reward for you in the thanks of your readers, but in any case, you have done a noble thing in helping the weak against one of the most impudent, useless, and mischievous superstitions that have ever been imposed on the helpless, innocent, and suffering children of men. Is the profession never going to ask to be relieved of the odium of the penal laws it has ignorantly imposed upon us?

I am yours truly,

ALEX. WHEELER.

